# Metastatic recurrent giant orbital ameloblastoma: A rare case report and literature review

**DOI:** 10.1097/MD.0000000000043348

**Published:** 2025-08-08

**Authors:** Rui Zhang, Xiaoming Huang, Yandi Huo, Rui Xie, Anshi Du, Tong Wu, Fengyuan Sun

**Affiliations:** a Department of Orbital Disease and Oculoplastic Surgery, Aier Eye Hospital (East of Chengdu), AIER Eye Hospital Group, Chengdu, Sichuan, China; b Department of Orbital Disease and Oculoplastic Surgery, Sichuan Eye Hospital, AIER Eye Hospital Group, Chengdu, Sichuan, China; c Department of Ophthalmology, Chengdu University of Traditional Chinese Medicine, Chengdu, Sichuan, China; d Department of Pathology Laboratory, Chengdu Gaoxin Da’an Medical Laboratory Company, Chengdu, Sichuan, China.

**Keywords:** case report, metastases, orbital ameloblastoma, rare giant orbital tumors, recurrent

## Abstract

**Rationale:**

Orbital ameloblastoma is a rare benign tumor with metastatic potential, typically exhibiting follicular or plexiform histopathological patterns. Ameloblastoma commonly occurs in the jaws and rarely metastasizes; when it does, the lungs and lymph nodes are the most frequent secondary sites. In the world’s reported literature, there are 32 cases of maxillary ameloblastoma metastasizing to the orbit, with only 4 cases of mandibular ameloblastoma metastasizing to the orbit. The mystery lies in its high recurrence rate and aggressive malignant potential despite being classified as a benign tumor, posing a serious threat to the ocular health and quality of life of patients.

**Patient concerns:**

This article reports an unusual case of a female patient who was initially diagnosed with mandibular ameloblastoma 17 years ago. Despite undergoing treatment, the tumor recurred and unusually metastasized to the orbit, resulting in a massive lesion that compressed the tissues surrounding the eyeball. She complained of gradual vision loss in her right eye, redness of the eye, and incomplete eyelid closure.

**Diagnoses:**

Magnetic resonance imaging (MRI) scans of the orbit indicated a lesion measuring approximately 5.8 cm*5.1 cm*5.7 cm (centimeter, cm). The clinical diagnosis is recurrent ameloblastoma of the right orbital, with the histopathological subtype being the basal cell type.

**Interventions:**

The patient underwent 2 successful partial excisions of the orbital tumor, effectively relieving the compression on the eyeball caused by the tumor.

**Outcomes:**

Currently, the 22-month follow-up after the second surgery has shown satisfactory results, with the patient’s visual function being preserved.

**Lessons:**

The patient exhibited significant facial disfigurement at the time of presentation to the ophthalmology department, attributable to the large size of the orbital mass. Inadequate awareness of this uncommon pathology may result in misdiagnosis as basal cell carcinoma or other malignant orbital neoplasms. Despite the preservation of visual function, limited understanding of the disease could lead to overly aggressive surgical management. Therefore, this case is reported to provide insights into the diagnosis, management, and prognosis, serving as a reference for clinicians encountering similar presentations.

## 1. Introduction

Orbital ameloblastoma is a rare, slowly progressing, benign tumor with metastatic potential, typically arising from the metastasis of residual odontogenic epithelium originating in the jaws to the orbit. Ameloblastoma is a benign, locally aggressive epithelial tumor arising from enamel, dental follicles, periodontal ligaments, or the linings of odontogenic cysts. They are the second most common odontogenic tumor, constituting 1% of all oral tumors and around 9% to 11% of odontogenic tumors. The World Health Organization (2017) classifies ameloblastomas into 4 types: conventional ameloblastoma (formerly termed “solid/multicystic ameloblastoma”), which includes follicular, plexiform, acanthomatous, desmoplastic, granular cell, and basal cell variants, either singly or in combination; unicystic ameloblastoma, with luminal, intraluminal, and mural variants; extraosseous/peripheral ameloblastoma; and malignant or metastatic ameloblastoma.^[1]^ The global incidence rate of ameloblastoma is 0.92 cases per million person-years, with a slightly higher incidence in males (53%).^[2]^ Malignant transformation and metastasis are extremely rare.^[3]^ Even if metastasis occurs, the most common secondary metastatic sites are the lungs and lymph nodes,^[4]^ with rare instances of tumor metastasis to the orbit.

Although orbital ameloblastoma is classified as a benign tumor, its high recurrence rate and malignant potential characterized by high invasiveness to surrounding tissues have posed challenges for treatment. Until recently, the main treatment for orbit ameloblastoma has been surgical resection, while other therapies, such as radiotherapy, chemotherapy, and molecular targeted therapy, have demonstrated limited effectiveness. Traditionally, due to the high invasiveness of ameloblastoma into surrounding bone tissue, surgical treatment should involve resection at least 5 millimeters beyond the tumor invasion site (including bone) to prevent recurrence.^[5,6]^ Incomplete treatment may lead to recurrence, and multiple recurrences can have a tendency towards malignant transformation. As malignant transformation is relatively rare, conservative surgeries that maximize the preservation of normal tissue, such as curettage, have been advocated by some experts in recent years.^[7–9]^

This case report highlights a significant research gap in understanding orbital ameloblastoma, particularly its rare metastatic potential despite being classified as a benign tumor. With only 4 reported cases of mandibular origin compared to 32 originating from the maxilla, this scarcity of data emphasizes the need for further research into the tumor’s behavior, treatment strategies, and clinical outcomes to enhance clinical management.

## 2. Case presentation

**Figure 1.**
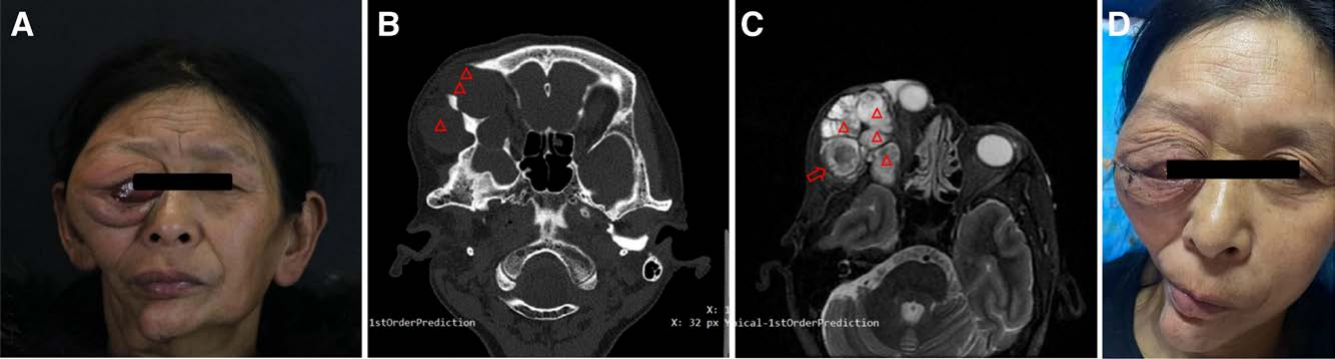
(A) The patient’s preoperative appearance. (B) Orbital CT (the tumor presents cystic changes, with the red triangle marking the tumor). (C) Orbital MRI (the red triangle marks the tumor, and the signal within the tumor cavity indicated by the red arrow is uneven). (D) The patient’s postoperative appearance. CT = computed tomography, MRI = magnetic resonance imaging.

In August 2021, a 56-year-old female from the Yi ethnic group in China presented with a diagnosis of ameloblastoma in the right orbit, and she had discovered a large tumor around her right eye socket 1 year prior. She underwent partial tumor resection surgical treatment for her condition. In February 2023, 18 months after surgery, the tumor in the same area began to gradually enlarge again, accompanied by a gradual decline in her right eye vision, eye redness, and incomplete eyelid closure. The patient did not experience any discomfort, such as headache, nasal congestion, or nasal bleeding. To address the issue of incomplete eyelid closure and relieve discomfort in her eye, she visited our hospital again (Fig. 1A–D). The patient had a history of smoking for more than 40 years and smoked approximately 20 cigarettes daily. In 2007, she was diagnosed with ameloblastoma in the right mandible and underwent surgical treatment. No radiotherapy or chemotherapy was administered after the surgery.

The ophthalmological examination on admission revealed the following: visual acuity was CF/20 cm (Counting Fingers, CF) in the right eye (uncorrectable), while the left eye maintained a visual acuity of 1.0. Intraocular pressure was within the normal range. A fixed, moderately hard mass was palpable in the superior and inferior regions as well as the temporal side of the right orbit. The mass had indistinct boundaries and extended from above the eyebrow to the mid-face, with prominent lateral protrusion. There was no pulsation or tenderness associated with the mass. Incomplete eyelid closure was observed, with a gap of approximately 3 mm, exposing part of the bulbar conjunctiva, which exhibited congestion and edema (+++). The globe of the right eye was displaced toward the nasal side, and its motility in all directions was suboptimal. Computed tomography (CT) scans of the orbit showed cystic changes in the tumor, with a maximum cross-sectional area of approximately 5.5 cm*4.8 cm. The tumor had invaded the orbit, pterygoid process of the sphenoid bone, zygomatic process, temporal process of the zygoma, and orbital rim of the frontal bone (Fig. 1A–D). Magnetic resonance imaging (MRI) scans of the orbit indicated a lesion measuring approximately 5.8 cm*5.1 cm*5.7 cm, which had breached the lateral wall of the orbit and grew toward the temporal side. The lesion was adjacent to the lateral rectus muscle and displaced medially. The cystic mass exhibited inhomogeneous signal intensity (Fig. 1A–D).

**Figure 2.**
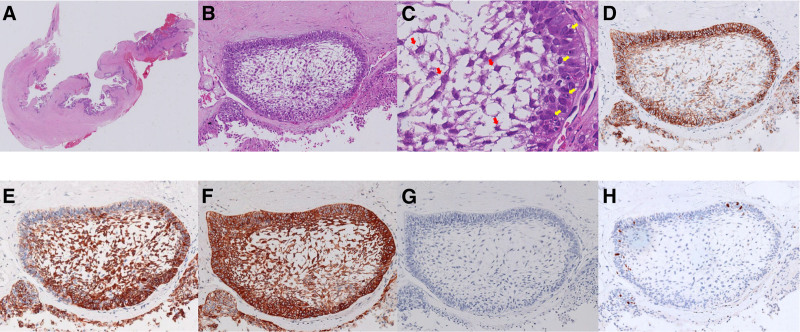
Pathological images of patients. (A) panoramic view (hematoxylin–eosin staining), (B) (enlarged ×100), and (C) (enlarged ×400). Two types of cells are visible under the microscope: 1 type is cuboidal or columnar (yellow arrow) and located at the periphery of the epithelial mass, with the nuclei arranged in a palisading pattern and away from the basement membrane; the other type is polygonal or stellate (red arrow) and located at the center of the epithelial mass, arranged loosely. Immunophenotype (magnification ×100): CD56(+) (D), CK19(+) (E), CK14(+) (F), BerEP4(−) (G). In differentiation from basal cell carcinoma, Kiel 67 shows low expression, approximately 3% (H).

We diagnosed her with recurrent ameloblastoma of the right orbit. Conservative medical treatment for this tumor is ineffective, and complete resection of the tumor poses a significant risk for facial reconstruction. Curettage is relatively safe and can address incomplete eyelid closure and local swelling sensations. After explanation and discussion with the patient and her family, our experts ultimately decided to perform curettage again. During the operation, we observed that the tumor was a large, irregular cystic mass with serous contents. We resected as much of the cyst wall as possible. The surgery was successfully completed. Postoperatively, the right eye globe retracted by 5 millimeters, the palpebral fissure height decreased by 6 millimeters, and the eyelids closed with a gap of approximately 2 millimeters, exposing a portion of the inferior bulbar conjunctiva. There was no corneal exposure, and visual acuity improved from CF/20 cm to 0.02. Eye movement improved in abduction, superior abduction, and inferior abduction compared to pre-operation, but movement was still suboptimal. There were no significant movement impairments in other directions. Pathological examination revealed ameloblastoma. Immunohistochemical staining was positive for CD56(Cluster of Differentiation 56, CD56), CK14 (Cytokeratin 14, CK14), CK19(Cytokeratin 19, CK19), and BerEP4(Epithelial Cell Adhesion Molecule Antibody, BerEP4). Kiel 67 was weakly positive, and calretinin was negative. Based on these findings, the subtype was considered the basal cell type (Fig. 2A–H). During a follow-up visit 1 year after surgery, the patient reported good eyelid closure, minimal residual swelling, and no signs of tumor progression. She was advised to visit the outpatient clinic regularly, and our doctors closely monitored any changes in the tumor.

This case obtained written informed consent from the patient for the publication.

## 3. Discussion

Ameloblastomas that originate in the mandible and eventually invade the orbit are indeed rare, with only 4 reported cases in the existing literature.^[10–13]^ These cases typically present initially with painless swelling in the maxillofacial area. As the disease progresses and invades the orbit, symptoms such as progressive protrusion of the eyeball, difficulty in eye movement, incomplete eyelid closure, and a gradual decline in vision may manifest. Invasion of the orbit and skull can occur over a span of 10 to 27 years after the initial diagnosis of mandibular ameloblastoma.

Two of these patients had the follicular type, while the pathological subtypes of the other 2 patients were not reported. The main treatment approach was as complete surgical resection as possible, supplemented with radiotherapy and chemotherapy techniques. In this case, the patient had a basal cell type orbital ameloblastoma and underwent curettage without the combined use of other therapies (Table 1). Residual tumor tissue after surgery is the main cause of recurrence, and misdiagnosis and mistreatment are also contributing factors to recurrence. CT is the most sensitive imaging method for diagnosing ameloblastoma.^[14]^ Early diagnosis of ameloblastoma is crucial for guiding treatment and preventing misdiagnosis.

**Table 1:** Four cases of mandibular ameloblastoma invading the orbit.

Author	Gender	Age	Patient nationality/region	Chief complaint	Recurrence (the number of recurrences)	Recurrence frequency/interval (yr) from initial diagnosis	After diagnosis, the orbit was invaded n years later	Infringement area	Treatment methods (order of treatment)	Outcome	Time from initial diagnosis to last follow-up (yr)
Moster Mark et al^[10]^	Female	37	Not mentioned	Double vision, left supraorbital pain	Progressive exophthalmos, gradual loss of visual acuity to no light perception, bulbar conjunctival swelling, and exposure of conjunctiva (1)	1 (4 mo after radiotherapy)	Not mentioned	Orbit, cavernous sinus, sella turcica, suprasellar	Craniotomy, biopsy (0); radiotherapy (1)	Death (cause: meningitis)	<1
Brazis et al^[11]^	Male	43	Not mentioned	Cystic lesions of the left mandible were found during dental examination	Welling of left upper gingiva (1); the left jaw angle was "plump" (2); bilateral horizontal diplopia with a small amount of vertical diplopia and sensory abnormalities in the left forehead (3); severe headache on the left forehead, numbness on the left upper face, dilation of the left pupil, complete ptosis of the left eye, disappearance of corneal reflex, recurrent blurred vision and diplopia in the left eye (4)	4 (4, 9, 10, 11)	10	Orbit, superior orbital fissure, cavernous sinus	Curettage (0); resection (1); radical resection (2); systemic steroid therapy, combined orbital/intracranial exploration (3); craniotomy (4)	Survival	10
Hayashi et al^[12]^	Female	63	Japan	Not mentioned	Swelling of the right lower gum (1); follow-up after surgery showed a shadow of bone absorption in the right mandibular bone (2); postoperative pathology revealed infiltrative tumor foci at the edge of surrounding tissues (3); rapid decline in vision in the right eye, central scotoma, and unequal size of pupils (right > left) (4)	4 (22, 25, 26, 27)	27	Orbit, extramandibular tissue, intracranial space, right frontal paralobular area	Mandibular resection (0); curettage (1); extralesional tumor resection (2); excision of lesion margin, postoperative radiotherapy and chemotherapy (3); radical resection (4)	Survival	27
Faras et al^[13]^	Female	56	Asia	Tenderness and swelling in mandibular region	The mandibular region is without tenderness and swelling (1); no mention was made (2); postoperative pathology indicates positive margins at the surgical site (3); and MRI suggests a heterogeneous mass behind the left eye that propels the left medial rectus muscle (4)	4 (19, 23, 23.25, 29)	23	Orbit, zygomatic arch, anterior wall of maxillary sinus, orbital floor, sphenoid wing	Mandibular resection (0); Surgical resection (1); Surgical resection + orbital floor reconstruction (2), lesion edge resection (3), surgical resection (4)	Survival	29
In this case	Female	56	Yi Nationality	Painless swelling of right mandible	A mass around the orbit (1); gradual visual loss in the right eye, eye redness, incomplete closure of the eyelid, suboptimal eyeball rotation, and MRI indicating the mass closely adheres to the lateral rectus muscle of the orbit and is pushed inward (2)	2 (14, 16)	14	Orbit, sphenoid pterygoid process, zygomatic process, zygomatic temporal process, frontal periorbital	Mandibular resection (0), partial resection of orbital ameloblastoma (1), partial resection (2)	Survival	17

MRI = magnetic resonance imaging.

Radical surgery is the preferred treatment for this disease, and ensuring complete resection of the tumor is a crucial step in reducing recurrence. However, when formulating a treatment plan, it is necessary to comprehensively consider the patient’s physical condition, his or her willingness to accept potential facial deformities, and his or her psychological expectations for postoperative quality of life. Despite being fully aware of the risk of recurrence if the tumor is not completely resected, we chose curettage for the following reasons: CT and MRI scans of this patient indicated that the recurrent tumor had invaded not only the orbit but also the sphenoid wing, the frontal bone around the orbit, the zygomatic process, and the temporal process of the zygoma. Radical surgery poses significant risks, such as cerebrospinal fluid leakage and optic nerve injury. Protection of cranial and facial functions and appearance is an important aspect of personalized treatment. Large-scale facial reconstruction is challenging, and even if successful, changes in the bones, muscles, and other tissues of the maxillofacial region may lead to dysfunction. This tumor has a low degree of malignancy, and conservative medical treatment is ineffective. The patient’s main complaint was local swelling and incomplete eyelid closure. If curettage is performed and the tumor recurs postoperatively, radical surgery can be postponed to a later stage of life.

The sole use of radiotherapy and chemotherapy for the treatment of ameloblastoma has shown limited effectiveness.^[12]^ Postoperative adjuvant radiotherapy provides the possibility of controlling the disease and delaying recurrence for patients with recurrent, positive resection margins or metastatic disease.^[15]^ However, there are insufficient data to support or reject the use of postoperative chemotherapy.^[16]^ In cases of metastasis, mutation-directed targeted therapy may offer additional salvage opportunities.^[17,18]^ Mandibular ameloblastoma is closely associated with BRAF gene mutations, while SMO gene mutations are more commonly observed in maxillary ameloblastoma.^[19]^ In our patient, despite the absence of adjuvant radiotherapy or targeted therapy, there was no shortening of the interval between recurrences.

In summary, ameloblastoma, while it generally has a low degree of malignancy, is relatively rare when it occurs in the orbit. Radical surgery is the preferred treatment approach. However, when the tumor involves crucial bony structures, the use of curettage should be carefully considered to avoid causing greater harm to the patient. Second, radiotherapy and molecular targeted therapy have achieved significant results in the treatment of this disease in recent years and can be considered perioperative adjuvant measures to prevent recurrence. Finally, after curettage, asymptomatic patients require continuous tumor monitoring, with at least annual clinical examination and CT scanning within 10 years postoperatively. For patients who are unwilling to comply with follow-up monitoring, radical surgery should be preferred to reduce the risk of recurrence.

## 4. Conclusions

This case report highlights the unusual presentation of metastatic recurrent giant orbital ameloblastoma and underscores the importance of maintaining a broad differential diagnosis in patients with orbital tumors. The rarity and aggressive behavior of this tumor emphasize the need for continued research and the development of targeted treatment strategies to improve patient outcomes. We hope that this report will contribute to the growing body of knowledge surrounding this unusual and challenging condition.

## Acknowledgments

We would like to express our gratitude to the patient for granting permission to use their clinical data in this paper and for the publication of this research.

## Author contributions

**Data curation:** Rui Zhang.

**Formal analysis:** Rui Zhang, Xiaoming Huang, Yandi Huo.

**Supervision:** Anshi Du, Tong Wu, Fengyuan Sun.

**Writing – original draft:** Rui Zhang, Xiaoming Huang, Yandi Huo, Rui Xie.
